# Tracheal airway pressure in tracheostomy tube capping trials: an experimental study

**DOI:** 10.1186/s12890-022-02277-4

**Published:** 2022-12-21

**Authors:** Andreas Nowak, Sten Martin, Maik Höhne, Winfried Heller, Taras I. Usichenko, Eckart Klemm

**Affiliations:** 1 Department of Anesthesiology and Intensive Care Medicine, Emergency Medicine and Pain Management, Dresden Friedrichstadt Hospital, Technical University Dresden Teaching Hospital, Friedrichstrasse 41, 01067 Dresden, Germany; 2 Faculty of Mechanical Engineering, University of Applied Sciences Dresden, Dresden, Germany; 3 Department of Anesthesiology, Intensive Care Medicine, Emergency Medicine, Pain Medicine, University Medicine of Greifswald, Greifswald, Germany; 4 Department of Anesthesia, McMaster University, Hamilton, Canada; 5 Department of Otorhinolaryngology, Head and Neck Surgery, Plastic Surgery, Dresden Friedrichstadt Hospital, Technical University Dresden Teaching Hospital, Dresden, Germany

**Keywords:** Tracheostomy, Tracheostomy tube, Decannulation, Capping trial, Cuff deflation, Stoma button

## Abstract

**Background:**

Tracheostomy tube capping is a commonly used test to determine if the tracheostomy tube can be removed. The success of the capping trial depends on the patient’s ability to maintain sufficient spontaneous breathing with an occluded tracheostomy tube. The impact of an occluded tracheotomy tube on airway resistance is currently unknown. The aim of this study was to investigate tracheal pressure during capping or stoma button insertion and potential determinants concerning cuff.

**Methods:**

Eight cuffed and uncuffed tracheostomy tubes and three stoma buttons of various manufacturers and sizes were inserted into the trachea model. Cuffs were completely deflated or contained atmospheric pressure. The trachea was ventilated bidirectional with a respirator in volume-controlled mode and volume flows 15–60 L/min. Tracheal pressure drop during inspiration as a parameter of pressure required to move gas through the airway was measured.

**Results:**

Tracheal pressure drops occurred linearly or irregularly during capping trials to a maximum of 4.2 kPa at flow rates of 60 L/min for atmospheric pressure cuffs. In tracheostomy tubes with completely deflated cuffs, pressure drop in the trachea reaches a maximum of 3.4 kPa at a flow rate of 60 L/min. For tracheostomy tubes with cuff smaller inner or outer diameters do not regularly result in lower tracheal pressure drop. The pressure drop varies between different tracheostomy tubes depending on the manufacturer. In cuffed tracheostomy tubes, we observed three phenomena: sail-like positioning, folding over, and tightening of the cuff during flow. The maximum tracheal pressure drop during stoma button insertion reaches 0.014 kPa.

**Conclusions:**

The cuff is a central element for the pressure drop in the airway and thus airway resistance during spontaneous translaryngeal breathing with a capped TT. Complete deflation reduces the pressure drop in the trachea. Due to deformation of the cuff, measured pressures are irregular as the volume flow is increased. Incomplete deflated cuffs and material characteristics of tracheostomy tubes and cuffs in addition to anatomical and clinical variables may cause unsuccessful capping trials due to increased airway resistance. All stoma buttons showed that pressure drop and thus airway resistance due to stoma buttons has no clinical relevance.

**Supplementary Information:**

The online version contains supplementary material available at 10.1186/s12890-022-02277-4.

## Background

In 2021, 36.087 temporary tracheostomies and 16.677 permanent tracheostomies were performed in Germany [1]. The National Confidential Enquiry into Patient Outcome and Death (NCEPOD) study estimated that approximately 12,000 adult tracheostomies a year were performed in England, Wales and Northern Ireland in 2014 [2]. Considering the underlying disease and the outcome of rehabilitation, a successful course of treatment leads to the decision of whether decannulation is possible. Requirements for successful decannulation include the following:

- clinical stability,
- sufficient spontaneous breathing,
- swallowing without aspiration,
- cough flow without the need for invasive secretion management,
- cooperation of the patient,
- no airway obstruction,
- cuff-leak test if necessary [3].

If the cuff-leak test is falsely positive, then there is a risk of unnecessarily prolonging ventilation. It is a well-known practice that successful decannulation is achieved even in patients who do not receive a cuff-leak test. There is no general consensus on the approach to decannulation and conversion to spontaneous breathing. In addition to spontaneous shrinkage of the tracheostoma after removal of the tracheostomy tube, stoma buttons (SB) or insertion of smaller diameter tracheostomy tubes (TT) can be applied [4]. Capping is a commonly used test to assess whether the TT can be removed. A systematic review has shown the use of capping for assessing readiness for decannulation in 63.7% of the patients [5]. However, capped TTs create an impairment of airflow [6]. Therefore, test decannulation using a SB may be performed [7]. Generally, the selection of methods is based on recommendations and experience with few objective data regarding pressures and flow conditions in airways [8]. The impact of deflated cuffs on airway resistance is currently unknown. The aim of this study was to investigate the pressure drop in the trachea as a variable of airway resistance due to capping or stoma button insertion and potential determinants such as the cuff and its behavior in terms of the volume flow. The pressure drop represents the pressure required to move gas through the airway around capped TTs or inserted SBs.

## Methods

**Fig. 1:**
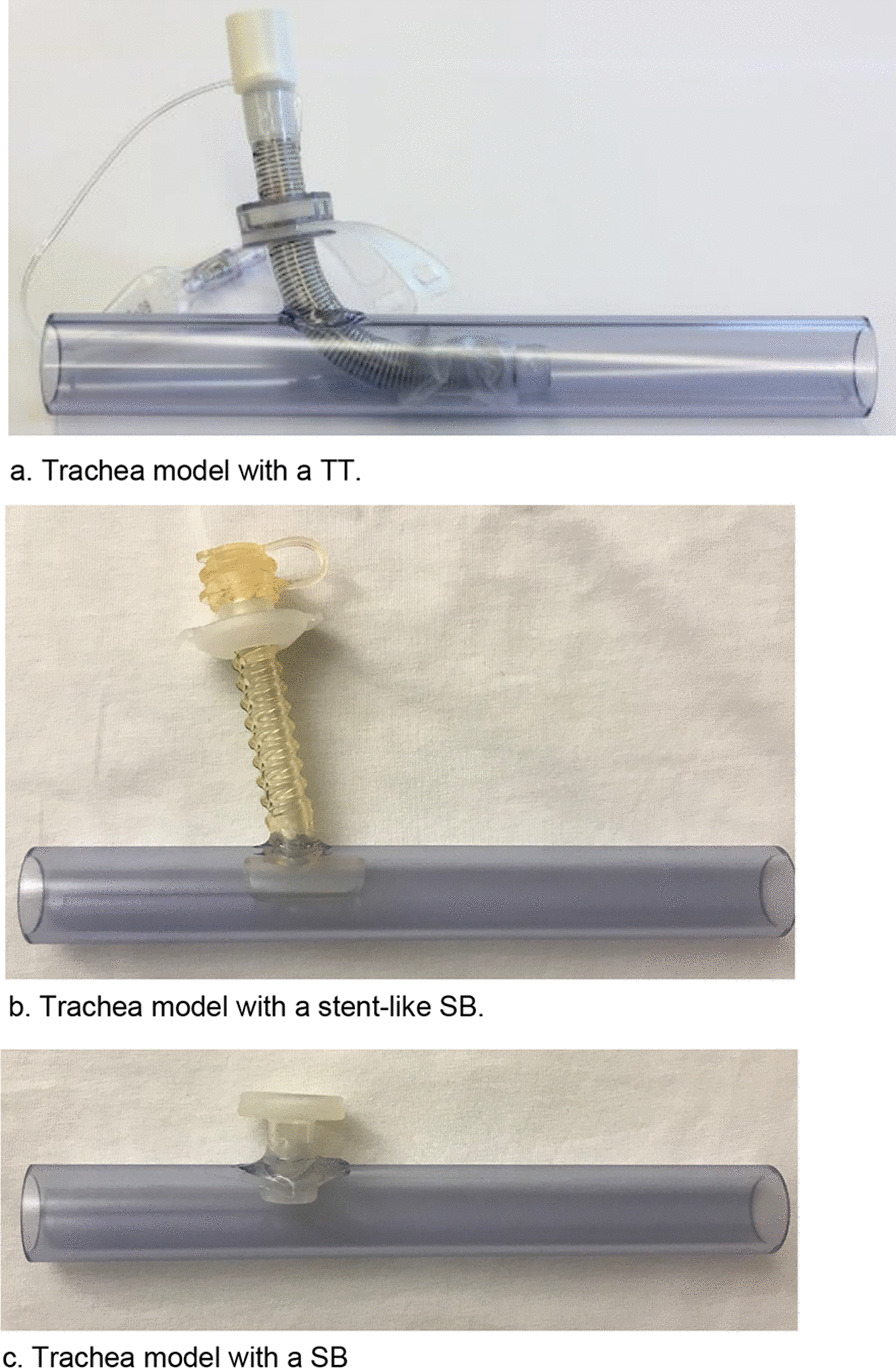
**a** Trachea model with a TT. **b** Trachea model with a stent-like SB. **c** Trachea model with a SB

**Fig. 2:**
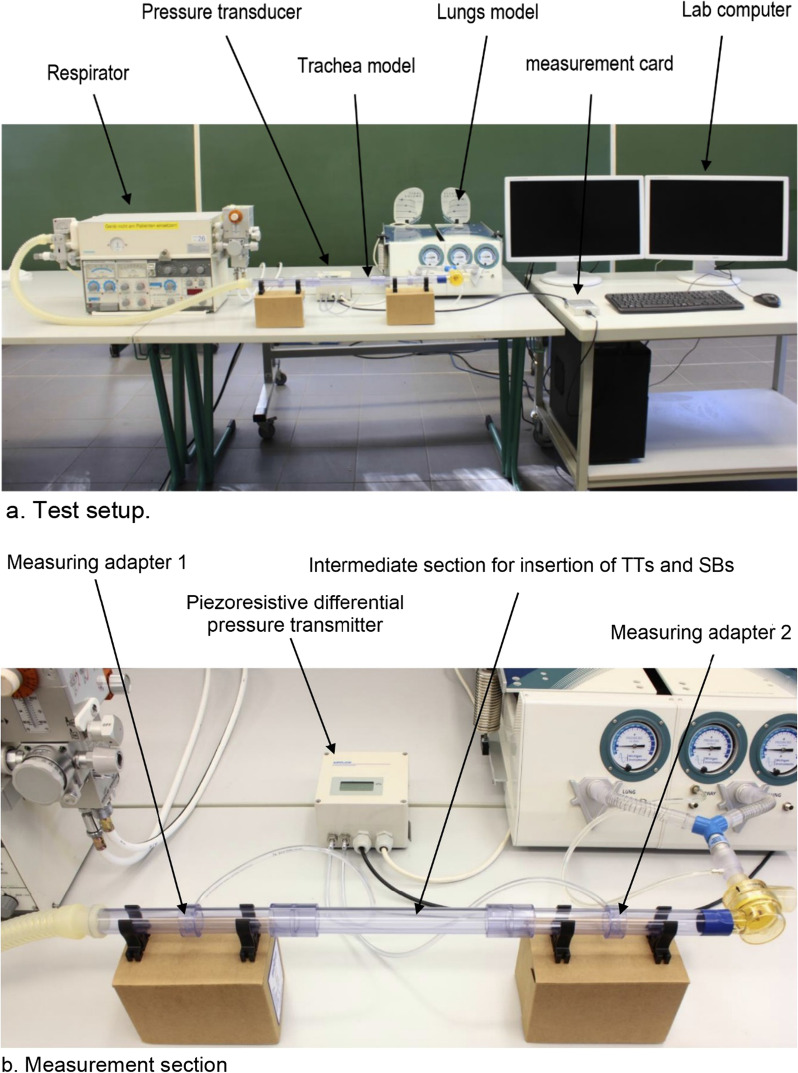
**a** Test setup. **b** Measurement section

Cuffed and uncuffed TTs of various models and sizes as well as SB were inserted into the tracheal model (Table 1) (Fig. 1a–c). The pressures during inspiration both above and behind the various TT or SB in the artificial trachea and thus the pressure drops were measured (Fig. 2a, b) (Additional file 1: Fig. A2). Twenty-four test series with TTs and SBs of different sizes and from different manufacturers were performed.

**Table 1:** TT and SB (manufacturer A–E)

Tracheostomy tube	ID* (mm)	OD** (mm)	Cuff	Cuff diameter (mm)	Fenestration
TT-1A-9C	9.0	12.2	+	34.0	−
TT-2B-9C	9.0	13.3	+	30.0	−
TT-3C-9C	9.0	12.1	+	30.0	−
TT-4D-9C	9.0	12.3	+	30.0	−
TT-5D-9C	9.0	12.3	+	30.0	−
TT-8C-9	9.0	12.1	−	n.a	−
TT-6B-7C	7.0	10.5	+	24.0	−
TT-7D-7C	7.0	9.7	+	26.0	−
SB-3D-9	9.0	12.0	n.a	n.a	n.a
SB-2D-7	7.0	10.0	n.a	n.a	n.a
SB-1E-7	7.0	10.0	n.a	n.a	n.a

*Inner diameter

**Outer diameter

**Fig. 3:**
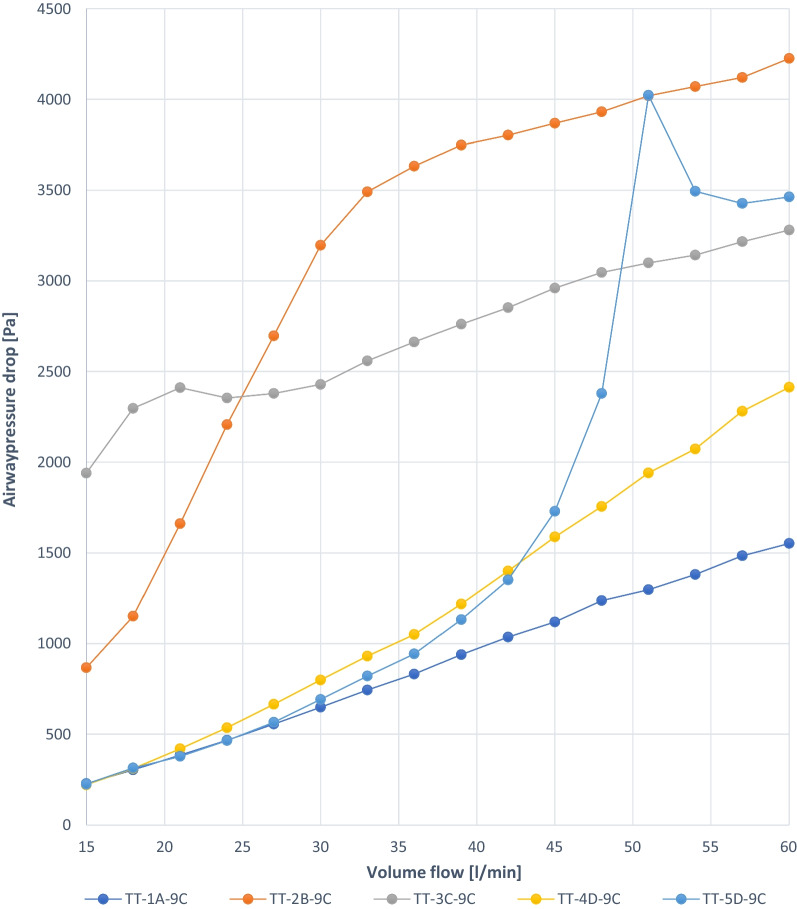
Experiment 1a: TT capped, similar ID 9 mm, deflated, different manufacturers (A–D)

**Fig. 4:**
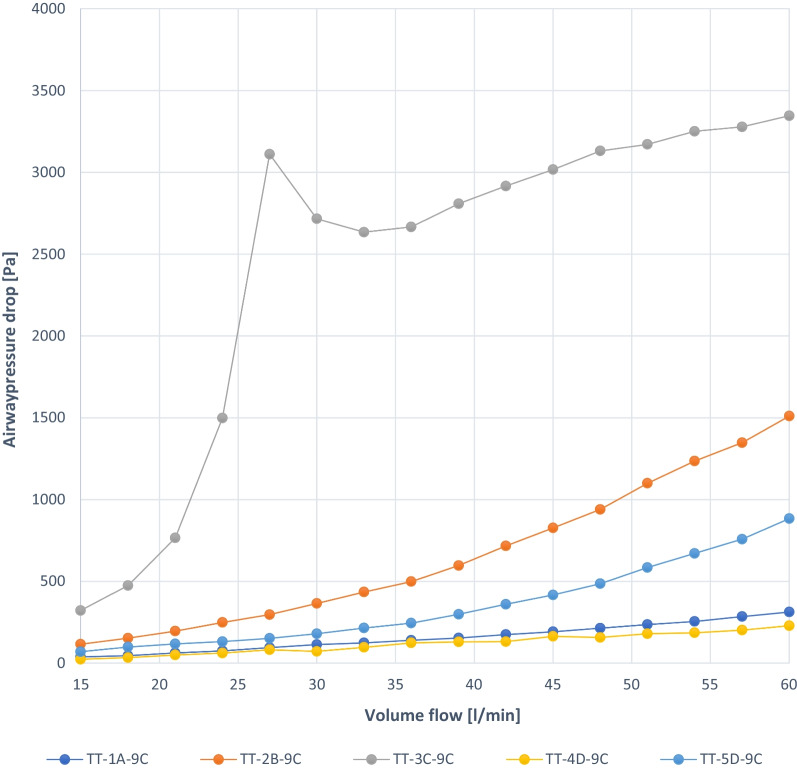
Experiment 1b: TT capped, similar ID 9 mm, deflated completely, different manufacturers (A–D)

**Fig. 5:**
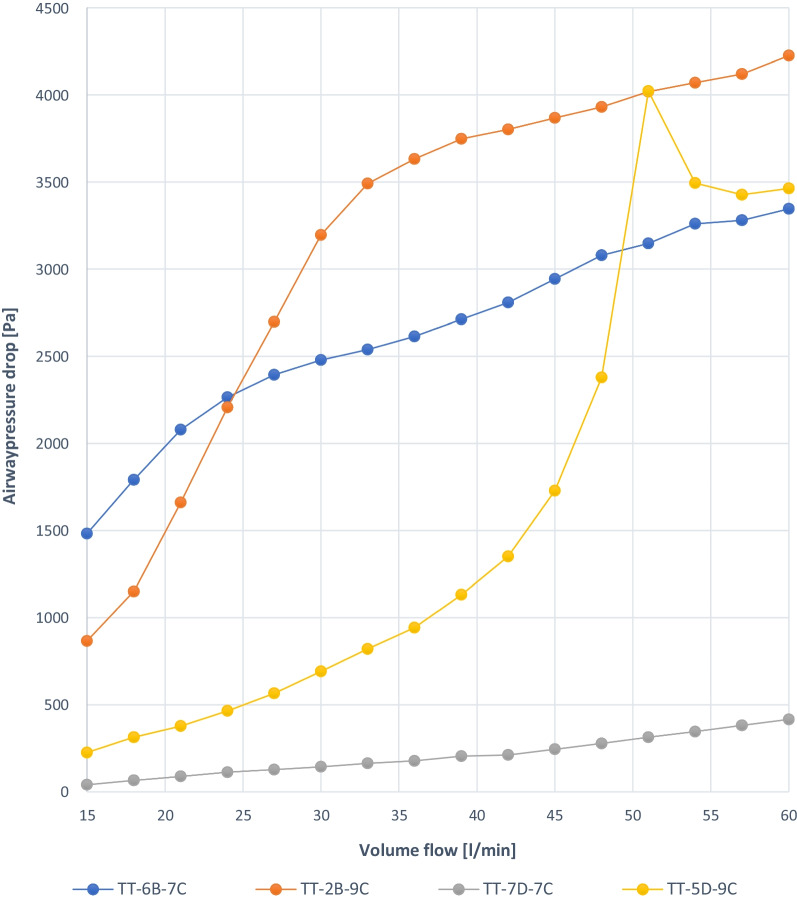
Experiment 2a: TT capped, ID 7 mm and ID 9 mm, deflated, similar manufacturer each (B, D)

**Fig. 6:**
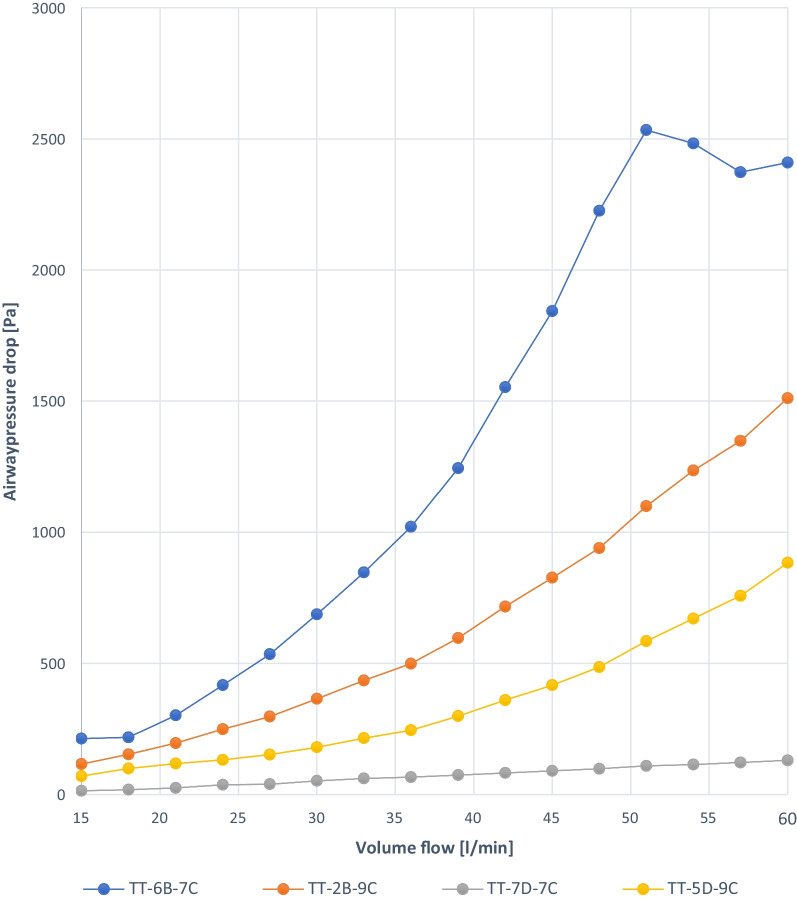
Experiment 2b: TT capped, ID 7 mm and ID 9 mm, deflated completely, similar manufacturer each (B, D)

**Fig. 7:**
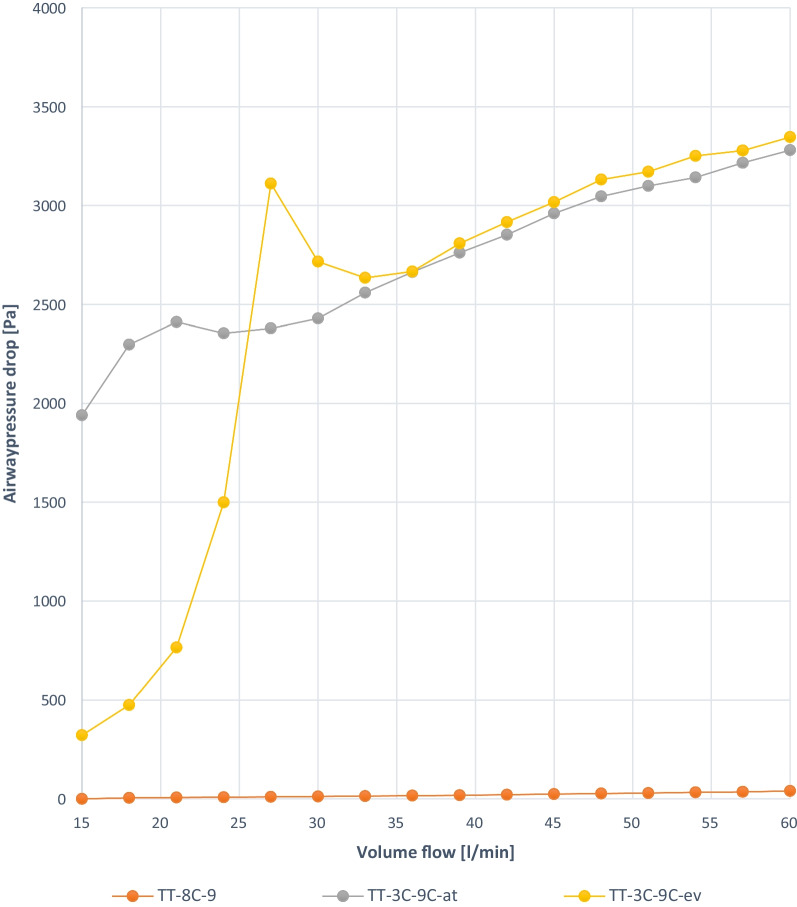
Experiment 3: TT capped, ID 9 mm with and without cuff, deflated [at] and deflated completely [ev], same manufacturer (C)

**Fig. 8:**
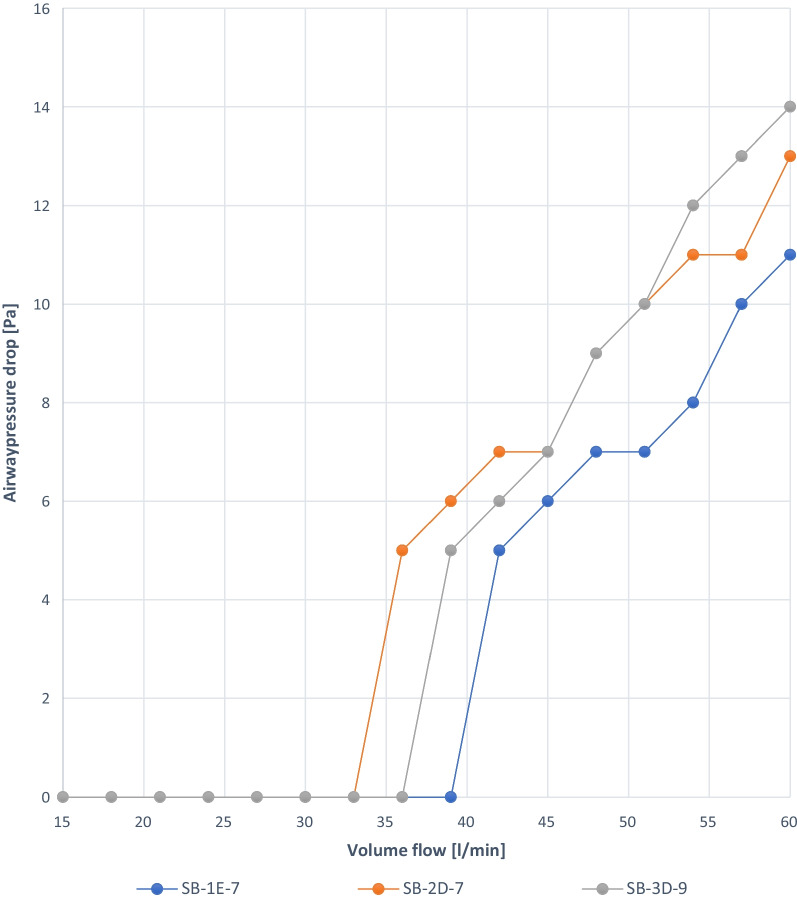
Experiment 4: Stoma buttons, different manufacturers (D, E)

For reasons of product neutrality, the tracheostomy tubes were coded in Table 1 and Figs. 3, 4, 5, 6, 7 and 8:

TT: Tracheostomy tube
SB: Stoma button
1–8: Number of tested tracheostomy tubes or stoma buttons
A–E: Manufacturer of the devices
7, 9: Inner diameter (ID) in mm
C: Tracheostomy tube with cuff

An adult training test lung (Michigan Instruments Inc., Grand Rapids, Michigan, USA) with an interfaced graduated trachea model (diameter 20 mm) was used in all experimental tests. The test lung was ventilated volume-controlled with a Siemens Servo Ventilator 900 C (Siemens-Elema, Gothenburg, Sweden) with a minute ventilation of 5–20 L/min [respiratory rate 20/min, inspiratory duration 33%; compliance of the ventilated test lung: 0.1 L/cm H_2_O] (Fig. 2a, b).

The reference measurement with a tracheal model without insertion of a tracheostomy tube or SB yielded a pressure drop in the complete volume flow range of less than 5 Pa. The calculated pressure drop due to pipe friction in the measuring section is 4.85 Pa. The pipe friction can be ignored. There were no errors in the measurement system (Additional file 1).

Data collection of over 30 respiratory cycles each consisting of inspiration and expiration for each sample was performed using LabView software (National Instruments, Austin, TX, USA) with a sampling frequency of 10 Hz. For each measurement, the sample size was n = 30. The raw data were mathematically processed. This process considered the random errors of the experimental study. Data were recorded by LabView software in an Excel file (Excel 2019, Microsoft Corporation, Redmond, WA, USA). The arithmetic mean was calculated, which was subsequently supplemented by a calculation of the empirical standard deviation. Since the mean values of a sample were also scattered, the measurement uncertainty was determined. For this purpose, a confidence level of 95% was applied. After calculating the arithmetic mean, standard deviation and measurement uncertainty, the complete measurement results were calculated for each measurement series (Additional file 1).

Experiments 1–4 were performed to investigate the pressure drop in the trachea due to capping or stoma button insertion and potential determinants concerning the cuff, as well as to compare between different ID, OD and manufacturers (Tables 2, 3, 4, 5, 6, 7). For the measurements of TTs with a cuff, a differentiation was made between the atmospheric cuff pressure and completely deflated cuff pressure. Atmospheric cuff pressure means that a positive pressure in the cuff has been released with residual air remaining in the cuff. The pressure in the cuff corresponds to the ambient atmospheric pressure. Completely deflated cuff pressure means that the cuff has been completely deflated with a syringe without residual air remaining in the cuff.

**Table 2:** Experiment 1a: TT capped, similar ID 9 mm, deflated, different manufacturers (A–D)

Experiment 1a	
TT cuffed—similar ID 9 mm different manufacturers (A-D)	Volume flow 15–60 L/min
Deflated, with atmospheric pressure	
TT-1A-9C	
TT-2B-9C	
TT-3C-9C	
TT-4D-9C	
TT-5D-9C

**Table 3:** Experiment 1b: TT capped, similar ID 9 mm, deflated completely, different manufacturers (A–D)

Experiment 1b	
TT cuffed—similar ID 9 mm different manufacturers (A–D)	Volume flow 15–60 L/min
Deflated completely, without residual air remaining in the cuff	
TT-1A-9C	
TT-2B-9C	
TT-3C-9C	
TT-4D-9C	
TT-5D-9C

**Table 4:** Experiment 2a: TT capped, ID 7 mm and ID 9 mm, deflated, similar manufacturers each (B, D)

Experiment 2a	
TT cuffed—ID 7 mm and ID 9 mm—manufacturer B	Volume flow 15–60 L/min
Deflated, with atmospheric pressure	
TT-6B-7C	
TT-2B-9C	
TT cuffed—ID 7 mm and ID 9 mm—manufacturer D	
Deflated, with atmospheric pressure	
TT-7D-7C	
TT-5D-9C

**Table 5:** Experiment 2b: TT capped, ID 7 mm and ID 9 mm, deflated completely, similar manufacturers each (B, D)

Experiment 2b	
TT cuffed—ID 7 mm and ID 9 mm—manufacturer B	Volume flow 15–60 L/min
Completely deflated, without residual air remaining in the cuff	
TT-6B-7C	
TT-2B-9C	
TT cuffed—ID 7 mm and ID 9 mm—manufacturer D	
Completely deflated, without residual air remaining in the cuff	
TT-7D-7C	
TT-5D-9C

**Table 6:** Experiment 3: TT ID 9 mm with and without cuff, deflated and deflated completely, same manufacturers (C)

Experiment 3	
TT uncuffed—ID 9 mm—manufacturer C	Volume flow 15–60 L/min
TT-8C-9	
TT cuffed—ID 9 mm—manufacturer C	
Deflated, with atmospheric pressure	
TT-3C-9C	
TT cuffed—ID 9 mm—manufacturer C	
completely deflated, without residual air remaining in the cuff	
TT-3C-9C

**Table 7:** Experiment 4: Stoma buttons, different manufacturers (D, E)

Experiment 4	
SB—ID 7 mm different manufacturers (D, E)	Volume flow 15–60 L/min
SB-2D-7	
SB-2E-7	
SB—ID 9 mm manufacturer D	
SB-3D-9	

## Results

### Experiment 1a

The graphs show linear pressure drops in the trachea up to more than 3 kPa for 3 TT (TT-1A-9C, TT-4D-9C, TT-3C-9C). With TT-2B-9C, the initial pressure drop was approximately 1 kPa. The airway pressure drop to approximately 3.5 kPa at flow rates up to 33 L/min and increases linearly to approximately 4.2 kPa at flow rates of 60 L/min. TT-5D-9C shows an irregular curve up to 4 kPa (Fig. 3).

### Experiment 1b

The graphs show almost linear small pressure drops in the trachea up to 1.5 kPa for 3 cannulas (TT-4D-9C, TT-1A-9C, TT-5D-9C, TT-2B-9C). TT-3C-9C shows an irregular pressure drop up to 3.4 kPa (Fig. 4).

### Experiment 2a

TT-7D-7C shows a linear pressure drop from 0.03 kPa at a volume flow of 15 L/min to approximately 0.4 kPa at a volume flow of 60 L/min. In comparison, TT-7D-7C demonstrates the lowest pressure drop if atmospheric pressure remains. In the 3 other cannulas, irregular pressure drops over the flow rate range of 15–60 L/min, regardless of the TT ID and OD (TT-6B-7C, TT-2B-9C, TT-5D-9C) (Fig. 5).

### Experiment 2b

TT-7D-7C shows an approximately linear pressure drop. At a volume flow of 15 L/min, a pressure of approximately 0.01 kPa was measured. Up to a volume flow of 60 L/min, the pressure drop increases to approximately 0.12 kPa. In comparison, the lowest pressure drop was measured with the TT-7D-7C if the cuff remained completely deflated. TT-5D-9C and TT-2B-9C show exponential graphs up to approximately 0.9 kPa and 1.5 kPa, respectively. For TT-6B-7C, the graph is irregular (Fig. 6).

### Experiment 3

The measurement results of TT-3C-9C are described in experiment 1.

TT-8C-9 without a cuff is characterized by a low approximately linear pressure increase. Below a volume flow of 18 L/min, the pressure increase is less than 0.005 kPa. At a volume flow of 60 L/min, the pressure increases to approximately 0.04 kPa (Fig. 7).

**Fig. 9:**
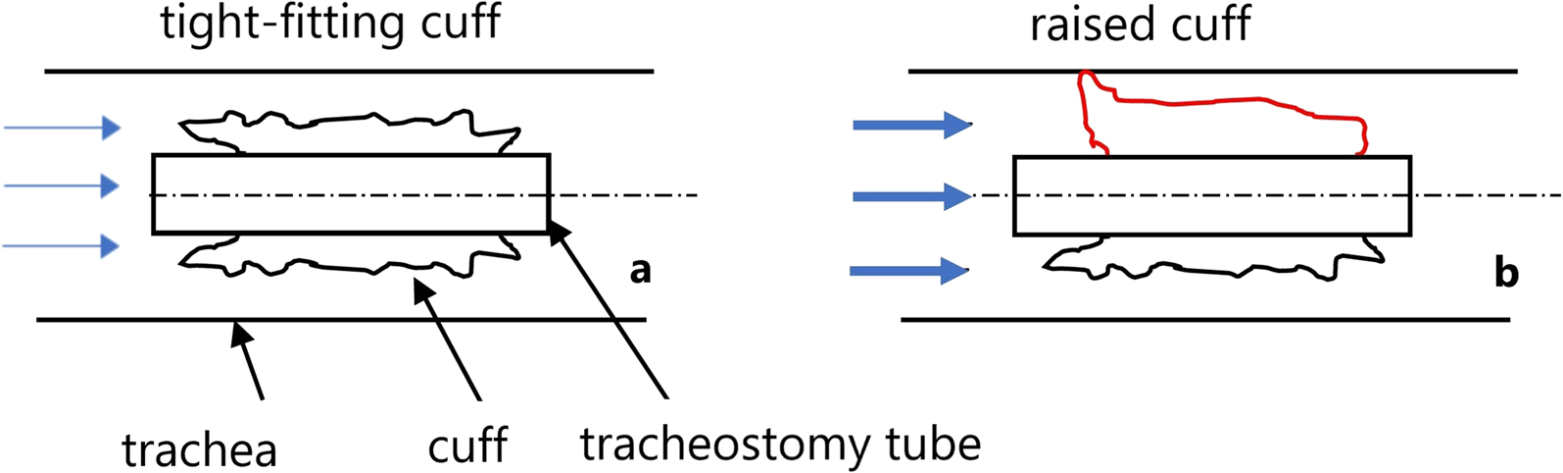
Sail-like positioning of the cuff in low (**a**) and higher (**b**) volume flows

**Fig. 10:**
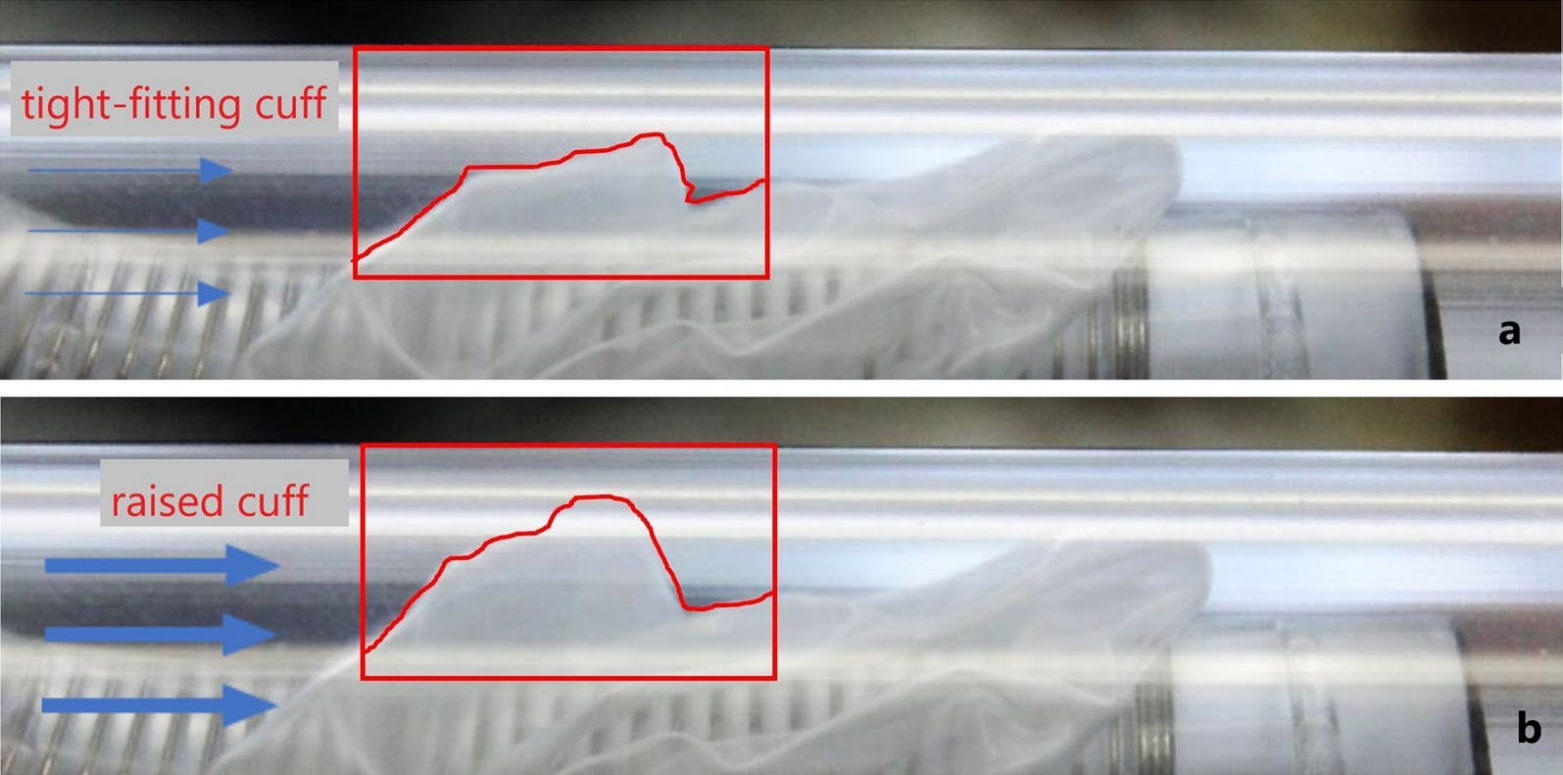
Sail-like positioning of the cuff in low (**a**) and higher (**b**) volume flows (experimental observation)

The pressure drop curves of the various TTs are different from each other. Theoretically, the pressure drop should rise exponentially when the flow velocity is increased. However, the measured results show different trends. This is because a sail-like deformation of the cuff occurs when the air flows around it (Figs. 9, 10).

### Experiment 4

Pressure measurements were performed on the SBs in the volume flow range of 15 to 60 L/min. In our experiments, small pressure drops were measured for the three stoma buttons SB-1E-7, SB-2D-7 and SB-3D-9. The maximum measured pressure drop for all SBs is 0.014 kPa, which has no practical relevance (Fig. 8).

### Deformation of the cuff due to the surrounding flow

Due to the deformation of the cuff during the flow, the measured pressure changes vary with changes in volume flow. Due to the transparent tracheal model, observations of the cuff could be made. Three phenomena were observed.

One of the most significant results is the sudden sail-like positioning of the cuff in the volume flow (phenomenon 1). In this case, the cuff is tightly attached to the tracheostomy tube at first. A minor increase in volume flow lifts the cuff from the tracheostomy tube and positions it in a sail-like shape in the trachea (Figs. 9, 10).

After the cuff has been set up and a breath has ended, the cuff either returns to the starting position or remains in the sail-like position. This phenomenon explains the sudden increase in pressure drop during the measurements.

**Fig. 11:**
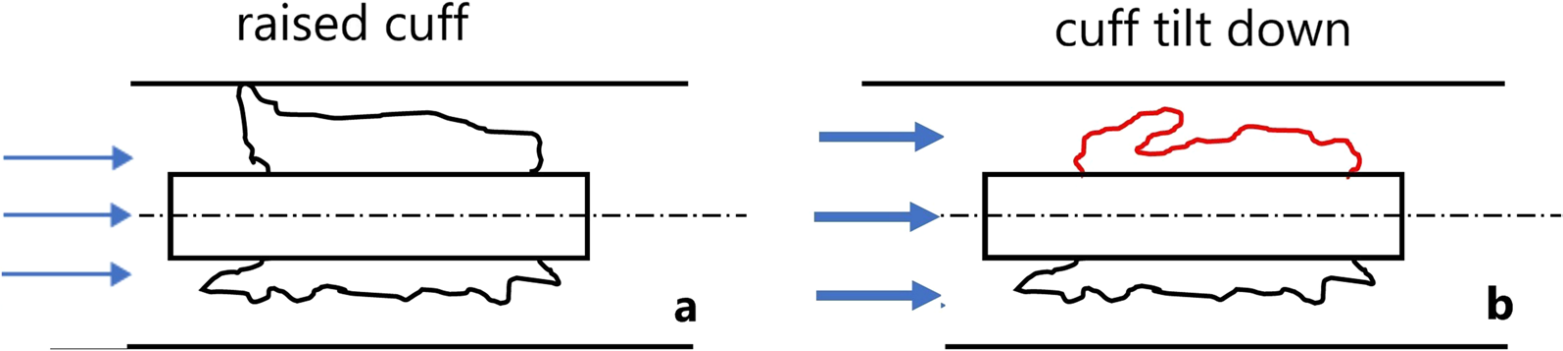
Raised cuff during low-volume-flow (**a**) and tilt down of the cuff during high-volume-flow (**b**)

A second phenomenon can be observed if the cuff remains in the setup position and the volume flow is further increased. The force equilibrium is disturbed, and the erected fold of the cuff tips occurs in the direction of the volume flow (phenomenon 2) (Fig. 11).

After the cuff is tipped over and the breath is completed, the fold of the cuff remains in this position. No repositioning was observed. In the curve of the pressure measurement, this phenomenon is visible as a sudden pressure drop decrease. The combination of sail-like positioning and tipping explains the peaks in the pressure diagrams.

**Fig. 12:**
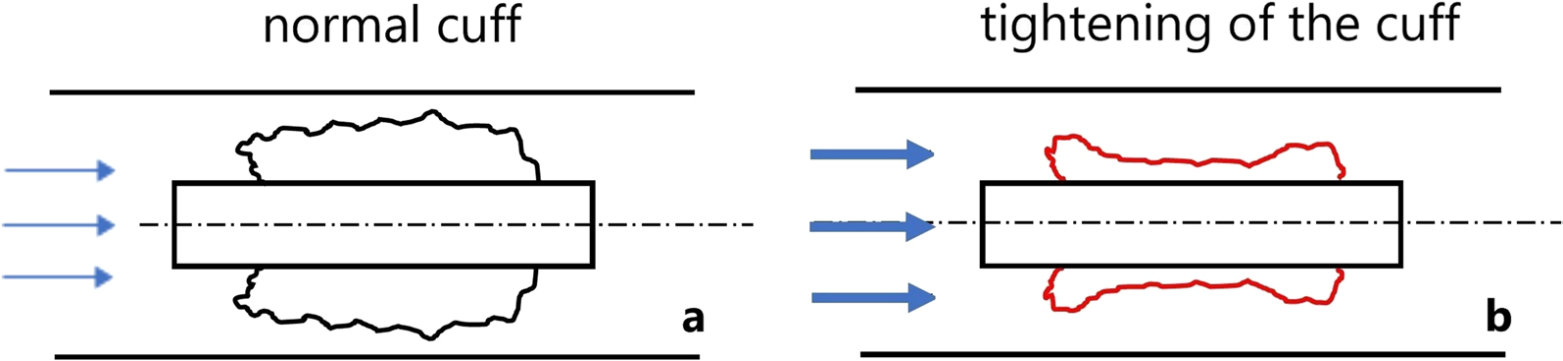
Tightening of the cuff during low flow (**a**) and with increasing volume flow rate (**b**)

The third observation is the tightening of the cuff during flow (phenomenon 3). This results in cuff material moving toward the cannula (Fig. 12). This movement was observed in higher volume flow ranges. A defined limit cannot be determined. After completion of a breath, the cuff returns to its initial position. In contrast to phenomena 1 and 2, the tightening of the cuff is not a sudden event. At the beginning of the next breath, the process starts again. It is difficult to see this phenomenon in the measurement results. The pressure drops do not increase exponentially but show only a weak exponential or linear increase.

These three phenomena, which can occur simultaneously, represent the new and important observations of our study.

## Discussion

Current prediction models for successful decannulation are based on algorithms using standardized TT capping [9]. According to our experimental results, capping generates pressures and flow conditions in airways that are highly variable depending on the selection of the TT and the patient’s respiratory minute volume.

In a clinical spirometry investigation, a dramatic airflow obstruction was found, mainly in expiration, due to occluded TTs during capping trials [6]. There are no standardized recommendations for decannulation and capping. To date, the algorithms for decannulation and the use of speaking valves have been based on patient factors [10]. According to our findings, the success or failure of existing algorithms is not only dependent on patient factors but is also determined technically. These findings have to be involved in the far-reaching decision of whether a TT can be removed. An deflated cuff, in which atmospheric pressure remains, always increases transtracheal pressures and thus the airway resistance in all TTs from different manufacturers. A deflated cuff that has been completely evacuated with a syringe that does not contain air produces lower airway resistance. Depending on the TTs and manufacturers with different material characteristics, these results are variable.

When capping a TT without cuff, ID 9.0 mm and OD 12.1 mm, an airway resistance of 0.36 kPa s l^−1^ is observed at a volume flow of 54 L/min, thus reaching the cutoff for mild obstruction (Fig. 7). All SBs of different manufacturers and sizes used in our study showed that airway resistance due to SBs has no clinical relevance. The number of successful decannulations will increase due to capping with a completely deflated cuff or TTs without cuff respectively insertions of SB. No clinical studies are available.

In the case of capping trial failure, we recommend:

Complete deflation of the cuff during capping.

When the capping trial is unsuccessful continuously, we recommend the following:

1. Consider change to a tracheostomy tube without cuff.
2. Application of a stoma button.

The TTs studied have outer diameters from 9.7 to 13.3 mm. Tracheostomy tubes with identical inner diameters (ID) may have different outer diameters (OD) according to the manufacturer (Tables 1, 2). No correlation between OD and pressure drop in the artificial trachea was found in our study.

The TTs studied have cuff diameters ranging from 24 to 34 mm. It is generally assumed that smaller cuff diameters create less resistance in the airway. No correlation was found in our study. In some cases, TTs with larger cuffs produce lower pressure increases than cannulas with smaller cuffs. In TTs with identical cuff diameters, the pressure increases vary widely. Across manufacturers, no reduction in airway pressure was observed when the cuff diameter was reduced. For TTs from an identical manufacturer and model series, the pressure drop was reduced with a decrease in the cuff diameter. The cuff diameter is not appropriate for a conclusion regarding the pressure drop in the trachea during capping.

**Fig. 13:**
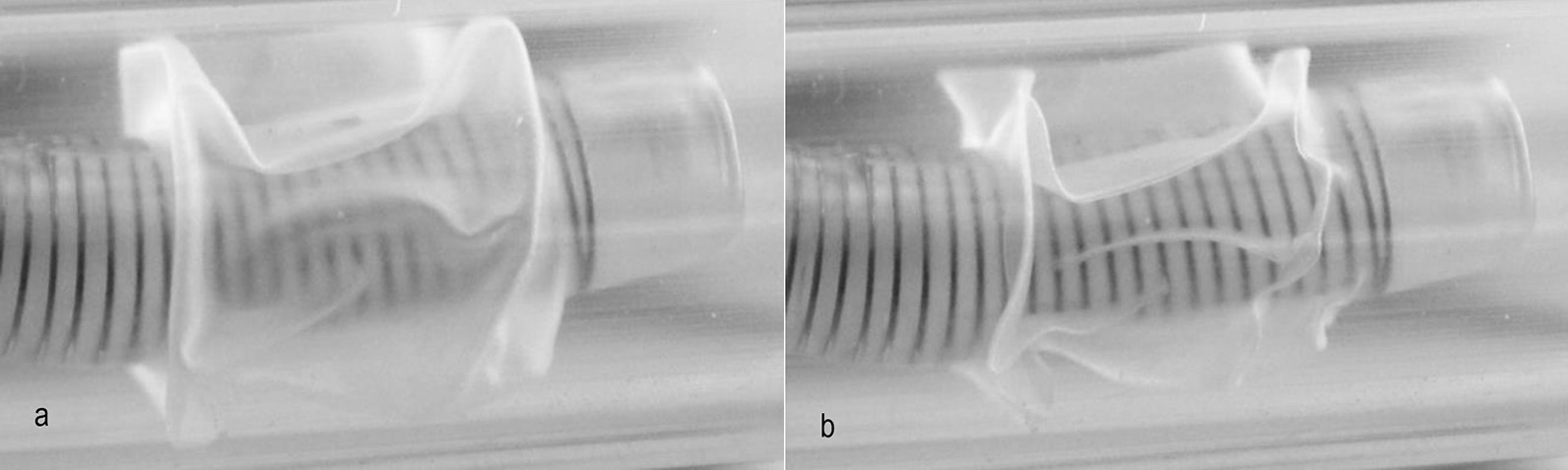
Deflated cuff with residual atmospheric pressure (**a**) and a completely deflated cuff (**b**)

A distinction is made between atmospheric and completely deflated cuff pressures for all TTs with cuffs. The cuff pressure of inflated TTs is usually measured to avoid pressure damage to the mucosa. In a deflated situation, less attention is given to the cuff pressure. Our study demonstrates that the cuff pressure increases the airway pressure during capping. In deflated TTs with an atmospheric cuff pressure, a higher pressure increase was observed. When the cuff was completely deflated, the pressure increase was reduced to 1/10^th^ of the value compared to the atmospheric cuff. Figure 13 illustrates the difference between an atmospheric cuff pressure with residual air remaining (Fig. 13a) and a completely evacuated cuff (Fig. 13b).

The conclusion for practice is that the cuff must be completely evacuated in the deflated state. With this small additional measure, spontaneous breathing during weaning from TTs can be made much easier for the patient. It does not escape our notice that complete deflation is not sustained due to diffusion of ambient air through the cuff membrane.

In our model the cuff is a central element for the pressure drop in the airway and thus airway resistance during spontaneous translaryngeal breathing with an capped TT. A description of the flow conditions with a mathematical model using numerical simulation (computational fluid dynamics (CFD) is feasible but requires the modeling of the different material characteristics of the cuffs. The material characteristics of the cuff influence its behavior in terms of the volume flow. For example, TK-3C-9C shows an inconsistent pressure graph despite complete deflation (Fig. 4).

When examining the flow patterns in the trachea, turbulent flows can be assumed. This type of flow is characterized by a three-dimensional flow field with an apparently randomly varying component in time and space. The three-dimensional flow can explain the tightening of the cuff (phenomenon 3).

### Limitations

There are limitations associated with our study. First, there could be a selection bias concerning the TTs that possibly influenced the significance of our findings. Second, the materials of the cuffs from the manufacturers differ. To investigate the impact on the pressure drop, the manufacturers were contacted. The responses showed that the three main materials used are polyvinyl chloride, silicone and polyurethane. Optical differences in the cuffs result from surface effects due to manufacturing. Not all manufacturers were willing to provide information on the cuff materials used. Due to incomplete information, no statements were made regarding the impact of the cuff material. For reasons of product neutrality, no information on the various manufacturers is provided in the study. Therefore, all devices tested were blinded. An answer to the question of which TT is particularly suitable for the weaning process cannot be given in this study. Third, in addition to our study, there may be numerous other reasons for failure of capping trials. These include mismatch of TT and trachea, secretions, granulations, obstruction of airflow in the upper airway, and others, also in combination. Our measurements were performed at room temperature and under dry conditions. Tracheal pressure drop and airway resistance may be higher in vivo due to higher temperature and moisture.

## Conclusion

With translaryngeal breathing, the pressure drop in the trachea due to capping of a TT with cuff is equivalent to severe airway obstruction and results in a considerable increase in airway resistance. Incompletely deflated cuffs with remaining atmospheric pressure and different material characteristics of tracheostomy tubes and cuffs induce irregular rather than the expected exponential pressure drops and may lead to unsuccessful closure attempts due to further increased airway resistance. When capping TTs without a cuff, mild obstruction is possible from high volume flows > 54 L/min. All SBs of different manufacturers and sizes used in our study showed that airway resistance due to SBs has no clinical relevance.

## Supplementary Information

**Additional file 1.** Measurement setup and calibration.

## Data Availability

The datasets collected and/or analyzed during the current study are available from the corresponding author upon reasonable request.

## Abbreviations

TT: Tracheostomy tube
SB: Stoma button
