# Supplementary material for: Tracheal airway pressure in tracheostomy tube capping trials: an experimental study
Source: BMC Pulm Med. 2022 Dec 21;22:484. doi: 10.1186/s12890-022-02277-4 (PMC9768925; doi:10.1186/s12890-022-02277-4)
Supplement: Supplementary file 1 — Additional file 1. Measurement setup and calibration. [file 12890_2022_2277_MOESM1_ESM.docx]

**Tracheal airway pressure in tracheostomy tube capping trials: An experimental study**

**Additional file 1**

**METHODS**

**Measurement setup**

***Trachea model***

A transparent polyvinyl chloride (PVC) tube was used as the trachea model. The inner diameter is 20 mm. The wall thickness of the tube is 2.5 mm, resulting in an outer diameter of 25 mm. The length is 200 mm. This allows observation of the inserted tracheostomy tube. Thus, the behavior of the cuff can be observed in its original position or during flow (Fig. A1).


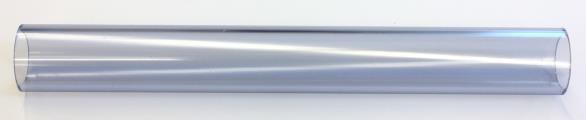


Fig. A1. Trachea model.

The opening for the tracheostomy tube or stoma button is located at 65 mm of the tube length. This means that the cuffs of the inserted tracheostomy tubes are approximately in the middle of the model. Each tracheostomy tube or stoma button was inserted into an individual tracheal model. After the tracheostomy tubes or stoma buttons were fitted into the tubes, the tracheal incisions were sealed with transparent silicone (Fig. 2).

***Pressure measuring adapter***

Two pressure measuring adapters of the PVC were inserted to register the static pressure above and below the tracheostomy tube or stoma button. These adapters are identical in construction to the PVC tubes of the tracheal models. This avoids cross-sectional transitions and unintended pressure losses. The pressure measuring points consist of four ports. These ports were connected to each other and joined to form an outgoing measuring line. The connection of the ports was created by a circumferential rectangular channel. A ring was fitted over the channel and glued in place. (Fig. A2).


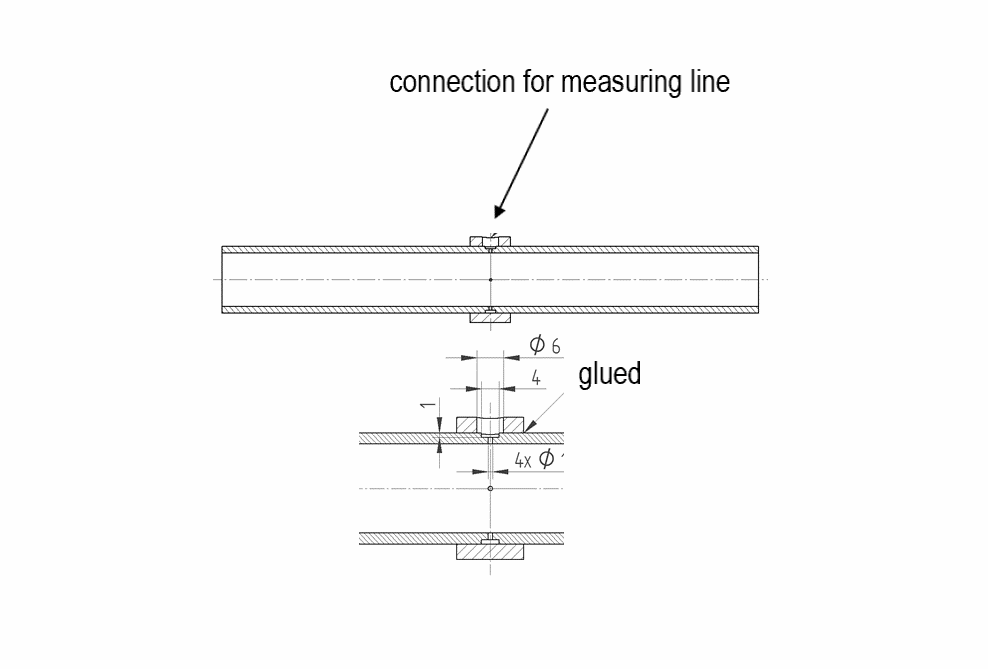


Fig. A2. Pressure measuring point in detail.

The measuring line was sealed airtight with a glued connection. The length of the measuring line is 0.5 m (Abb. A3).


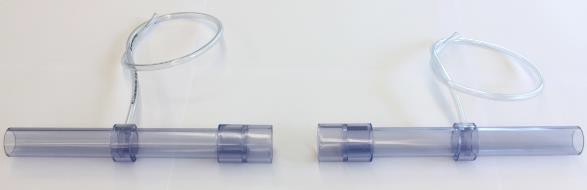


Fig. A3. Pressure measuring adapter with measuring lines.

***Pressure transmitter***

A piezoresistive differential pressure transmitter was used to measure the pressure difference between the two measuring lines (AIRFLOW Lufttechnik GmbH, Rheinbach, Germany) (Fig. A4). The measuring range of the transmitter is from 0 to 5 kPa.


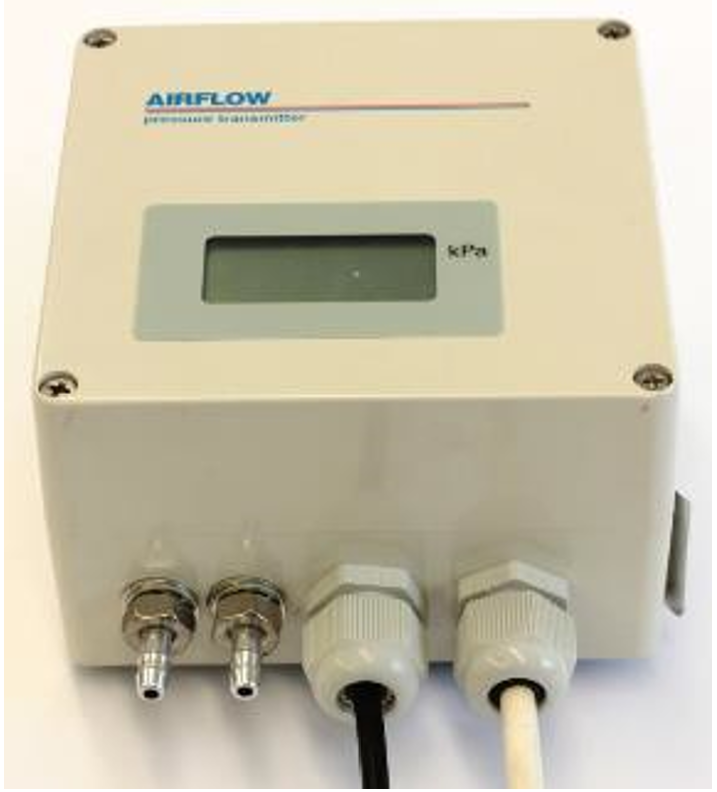


Fig. A4. Piezoresistive differential pressure transmitter.

***Measuring card***

The electrical signal of the piezoresistive differential pressure transmitter is sent to an analog/digital converter (A/D converter). This enables computer-assisted evaluation of the measurement data. An NI 9215 measuring card (NATIONAL INSTRUMENTS, Austin, Texas, USA) was used in conjunction with an NI USB 9162 Hi-Speed USB carrier (NATIONAL INSTRUMENTS, Austin, Texas, USA). The measuring card has four channels with a maximum measuring range of ± 10 V and a resolution of 16 bits (Fig. A5). The corresponding software LabVIEW was used to process and display the data on the computer (National Instruments, Austin, TX, USA).


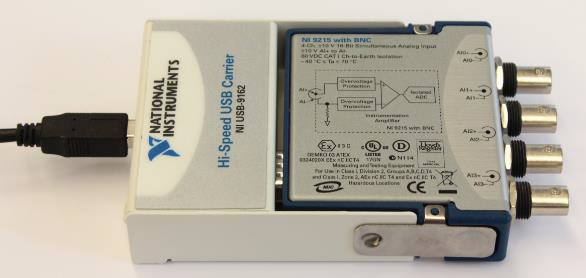


Fig. A5. Measuring card NI 9215 mit USB Carrier NI 9162 (NATIONAL INSTRUMENTS, Austin, Texas, USA).

***Ventilator***

An SV900 ventilator (Siemens-Elema, Gothenburg, Sweden) was used. This allows precise adjustment of the volume flow. A volume-controlled ventilation mode with constant volume flow during inspiration was used. This provided a square wave signal of the volume flow with constant amplitude. Only the relevant part with constant volume flow was processed by computer-assisted signal analysis (Fig. A6).


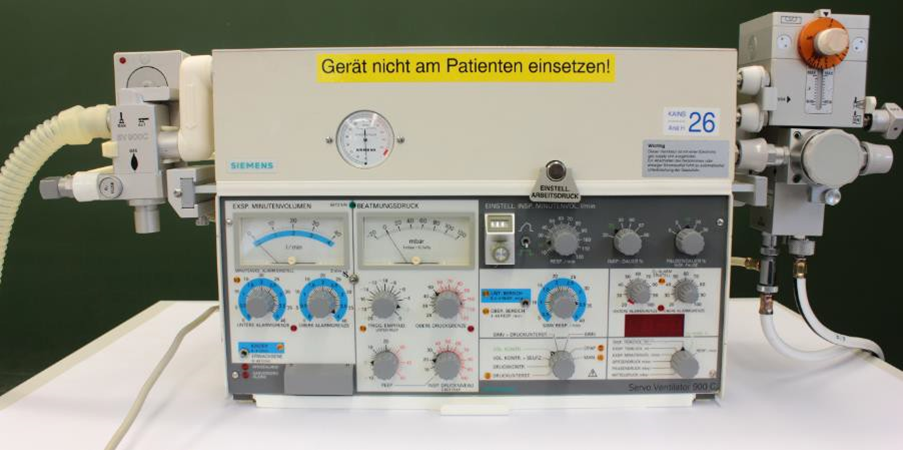


Fig. A6. Ventilator SV 900C (Siemens-Elema, Gothenburg, Sweden).

***Lung model***

In the experiment, the PneuView3 lung model (Michigan Instruments, Grand Rapids, MI, USA) was used to control the volumetric flow rate. The measured volumetric flow was visualized by PneuView3 software (Michigan Instruments, Grand Rapids, MI, USA). (Fig. A7).


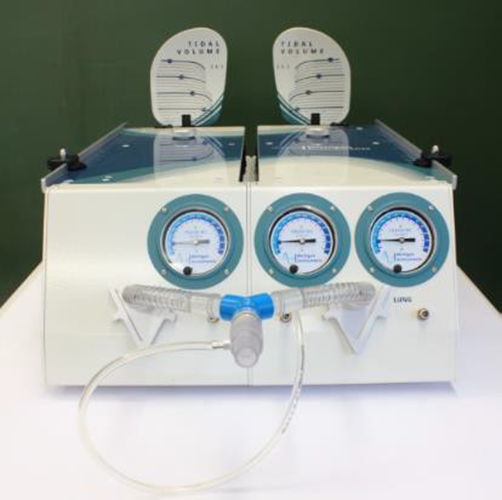


Fig. A7. Lung model PneuView3 (Michigan Instruments, Grand Rapids, MI, USA).

***Measurement section***

The SV900 ventilator (Siemens-Elema, Gothenburg, Sweden) was connected to the entry of the measuring section via a silicone hose. Subsequently, an empty tube without an inserted tracheostomy tube was installed as a reference. The lung model was connected to the outlet of the measuring section. After installation of the air conducting components, the pressure transmitter was connected to the two measuring lines of the pressure measuring adapters. The pressure transmitter was connected to the measuring card via the BNC connection 0 (Figs. A4 and 2b).

***Calibration***

A combination of calibration and adjustment was performed. In this process, the nominal-actual deviation was not only determined but also eliminated. The pressure transmitter was connected to an electronic pressure calibration system. This allows the setting of a defined test pressure, which corresponded to the nominal value. The measured voltage was stored in LabVIEW measuring software (National Instruments, Austin, TX, USA). The measured voltage represented the actual value. This procedure was carried out with a step size of 1 mbar over the measuring range. Finally, the correction of the nominal-actual deviation was performed within the software. The corrected losses include voltage losses through lines, plug connections and within the laboratory computer.

***Reference measurement with an empty tube***

The reference measurement with a tracheal model without installation of a tracheostomy tube or stoma button was used for the zero offset. This was used to check the pipe friction within the measurement section and possible further pressure losses. The aim was to determine the pressure losses that are not generated by tracheostomy tubes or placeholders. The measurements were performed in ascending steps of 3 l/min over a volume flow range of 15 l/min to 60 l/min. For each set volume flow rate, 30 ventilation trains (30 square wave signals) were recorded. The set frequency on the ventilator was 20 breaths per minute, which resulted in a measurement duration of 90 seconds. The Shanon-Nyquist theorem was considered during the measurement.

The sampling frequency was at least twice the frequency to be measured. The selected sampling frequency was 10 Hz, which is above twice the measurement frequency of approximately 0.33 Hz (20 breaths per minute). The reference measurement yielded a pressure loss in the complete volume flow range of less than 5 Pa. A mathematical check of the reference measurement was carried out using the formula for the pressure loss in a straight pipe [1]:


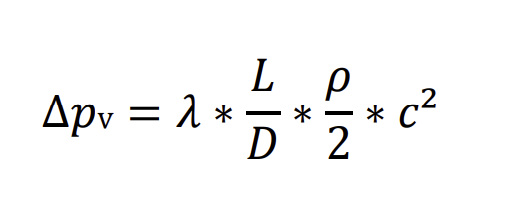


In straight pipes, the dimensionless loss coefficient ζ is replaced by the friction factor λ and the quotient of pipe length L and tube diameter D. For turbulent flow, the friction factor λ is a function of the Reynolds number Re and the ratio of the pipe diameter D to the pipe roughness k:


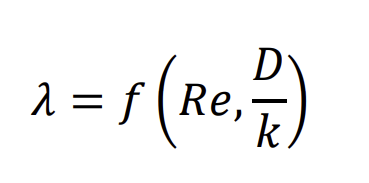


The friction factor was determined using the Nikuradse-Colebrook-Moody pipe friction diagram. The Reynolds number is defined by the formula:


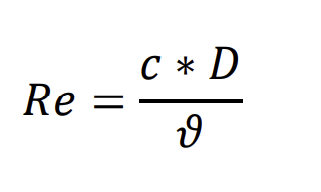


The pipe roughness k of plastic pipes is 0.002 mm [2]. The pipe diameter D is 20 mm. For the maximum volume flow of 60 l/min, the flow velocity was calculated using the continuity equation:


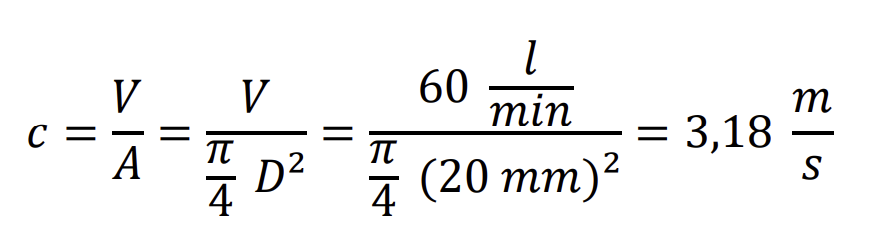


The kinematic viscosity ϑ of air is approximately 1.4*10-5 m²/s. This results in a maximum Reynolds number of

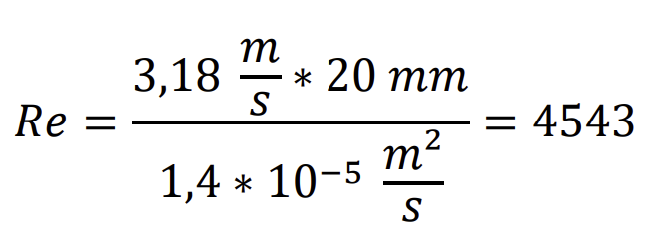


The transition from laminar to turbulent pipe flow occurs at a Reynolds number of approximately 2000 [1]. With a Reynolds number of 4543, turbulent pipe flow can be assumed. The ratio of pipe diameter D to pipe roughness k is
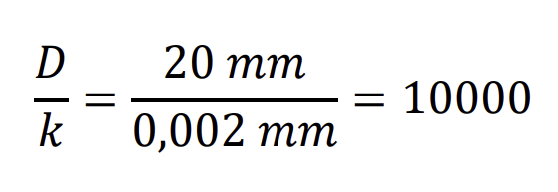


From the pipe friction diagram, the friction factor λ is

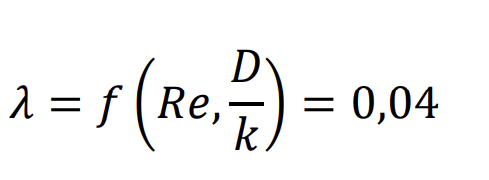


The tube length L between the two pressure measuring points is 400 mm. The density ρ of the air is 1.2 kg/m³ [3]. This is followed by a pressure loss of
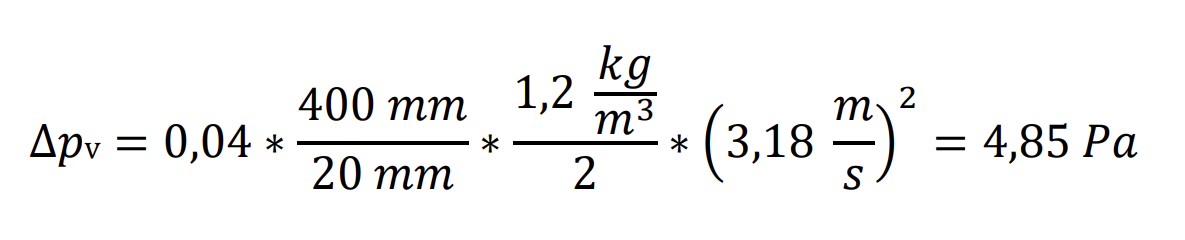


The calculated pressure loss due to pipe friction in the measuring section is 4.85 Pa. The pipe friction can be ignored. There were no errors in the measurement system.

**References**

1. Heller W. Zum Einfluss der Turbulentenz der Anströmung auf die Druckwirkungen in Grenzschichten und den Kavitationsbeginn. Dissertation. Technische Universität Dresden; 1997.

2. Schweizer FN. Rohrrauigkeit. 2019. <https://schweizer-fn.de/stroemung/rauhigkeit/rauhigkeit.php>. Accessed 27 Apr 2022.

3. Langenheinecke K, Jany P, Thieleke G, Langeheinecke K, Kaufman A. Thermodynamik für Ingenieure. Springer Fachmedien Wiesbaden. 2013. <https://link.springer.com/content/pdf/bbm%3A978-3-658-03169-5%2F1.pdf>. Accessed 27 Apr 2022.
